# Climate-related psychological distress among reproductive-age women: A qualitative content analysis

**DOI:** 10.1016/j.joclim.2026.100760

**Published:** 2026-10-28

**Authors:** Zahra Alipour, Maryam Ghaedrahmati, Faride Yousefizadeh

**Affiliations:** a Department of Midwifery, School of Medicine, Qom University of Medical Sciences, Qom, Iran; b PhD in Reproductive Health, Narges Social Security Organisation, Dorud, Lorestan, Iran; c Department of Nursing, Behbahan Faculty of Medical Sciences, Behbahan, Iran

**Keywords:** Climate change, Mental health, Women, Psychological distress, Qualitative research, Iran

## Abstract

**Introduction:**

Climate change can affect mental health through chronic environmental exposure and disruption of everyday life. Women may encounter these pressures through caregiving, household resource management, and concerns for family well-being. This study explored reproductive-aged women’s perceptions and experiences of climate-related psychological distress in Qom, Iran.

**Methods:**

Conventional qualitative content analysis was used. Twenty-five women aged 18–45 years were recruited from health centers, universities, parks, and other community settings using maximum-variation purposive sampling. Variations were sought in age, marital status, education, occupational status, and recruitment setting. Semi-structured interviews were conducted and analysed inductively in Persian. Saturation was reached after interview 17 and confirmed through eight additional interviews.

**Results:**

Four themes, nine categories, and 17 subcategories were developed: Living with a disrupted and increasingly insecure environment; consequences of climate change in everyday life; climate-related psychological distress; and social consequences of climate change for mental health. Participants linked heat, drought, water insecurity, salinity, dust, and air pollution with bodily discomfort; household and livelihood pressures; emotional responses such as fear, irritability, and place-based grief; cognitive preoccupation with caregiving, resources, and future liveability; moral self-monitoring during household water use; and family tension.

**Major conclusions:**

Climate-related psychological distress emerged as a cumulative, situated response with interrelated emotional, cognitive, and moral manifestations embedded in environmental, bodily, material, caregiving, relational, and place-based contexts. These responses should not automatically be pathologized. The findings support the co-design and evaluation of locally tailored responses that integrate psychosocial support with practical adaptation across primary care, university, and community settings.

## 1 Introduction

Climate change is an increasingly important determinant of population health, with consequences extending beyond environmental and physical harm to psychological, social, economic, and intergenerational well-being [1,2]. Mental health effects arise from direct exposure to heat, drought, and extreme events, as well as indirect pathways such as water and food insecurity, livelihood disruption, displacement, and pressure on health systems [1,2]. Importantly, these effects are not confined to acute disasters. Chronic, slow-onset changes, including prolonged drought, long-term temperature change, and perceived ecosystem degradation, have been associated with psychological distress and emotions such as worry, grief, sadness, and concern about the future [3,4].

Climate-related psychological distress may include anxiety and worry, sadness, helplessness, guilt, rumination, ecological grief, and uncertainty about the future [[5], [6], [7]]. These responses should not automatically be pathologized; anxiety, worry, and grief may be understandable and sometimes constructive responses to environmental threat and loss [5,7]. Nevertheless, when distress becomes sustained or disrupts relationships, daily functioning, or well-being, it becomes relevant to both mental and public health [2,5,7]. This non-diagnostic framing is particularly appropriate for qualitative inquiry, which can capture non-clinical emotional responses and contextualize how people experience chronic environmental change [3].

Climate-related psychological and social burdens are not distributed equally, and gender is central to understanding vulnerability [8,9]. Women may be particularly affected when environmental and economic pressures interact with gendered caregiving roles, household resource management, water and food insecurity, reproductive health, and unequal access to resources and services [8,9]. Among women of reproductive age, concerns may also extend to childbearing decisions, children's health, and the liveability of future environments [10,11]. Although not climate focused, a cross-sectional study of 853 adolescent girls in Qom identified substantial perceived unmet needs across psychological-emotional, social, physical, educational, counseling, and social-support domains [12].

Iran is an important setting for such inquiries. Studies in Iran have documented or projected changes in temperature and precipitation, with implications for drought, water availability, agriculture, farm livelihoods, and adaptive responses [13,14]. Near-term projections for 2023–2028 indicate increases in drought frequency and heat-wave activity across much of Iran, with extensive areas classified as having high or very high compound climate hazard levels [15]. Qom has an exceptionally dry climate, and meteorological records document its low precipitation [16]. A study in Qom brick workshops measured occupational heat exposure [17], while a separate building study simulated the effects of residential plan forms on thermal comfort in Qom [18]. In the Salt Lake catchment, including the Qom-Kahak and Salafchegan-Neyzar aquifers, population growth, agricultural and industrial development, and groundwater abstraction have contributed to declining groundwater levels and increasing water stress [19]. Taken together, these climatic and water-resource characteristics make Qom a relevant setting for examining how chronic environmental change is perceived and experienced in everyday life and mental health [[13], [14], [15], [16], [17], [18], [19]].

Despite the growing literature on climate change and mental health, important gaps remain. Compared with the extensive literature on acute disasters, the mental health effects of chronic, slow-onset climate change remain relatively under-researched, and quantitative studies have commonly assessed anxiety, depression, suicidality, and nonspecific psychological distress [3]. Evidence on women’s climate-related mental health remains limited, particularly in climate-vulnerable low- and middle-income settings, where heat and water insecurity may be important stressors [8,9,20]. Qualitative evidence from two climate-vulnerable communities in Bangladesh suggests that women’s psychological suffering is shaped by interacting environmental and socioeconomic determinants [20]. Although an Iranian qualitative study has examined how climate change influences reproductive decision-making among women and men of reproductive age [10], the broader everyday experience of climate-related psychological distress among reproductive-aged women in a dry Iranian city warrants further investigation. Conventional qualitative content analysis is appropriate for organizing interview data and inductively eliciting meaning from participants’ accounts [21]. In the present study, this approach was used to examine participants’ experiences without imposing predefined clinical symptom categories.

Accordingly, this study used conventional qualitative content analysis to explore how reproductive-aged women in Qom perceive and experience climate-related psychological distress in the context of chronic environmental change.

## 2 Materials and methods

### 2.1 Study design and setting

This qualitative study used conventional content analysis to explore reproductive-aged women’s perceptions and experiences of climate-related psychological distress in Qom, Iran. The study was conducted from May to August 2024 and is reported in accordance with the Consolidated Criteria for Reporting Qualitative Research (COREQ) [22]. A complete checklist is provided in Supplementary File 2.

Qom, a semi-arid city located in central Iran, is characterized by hot summers and low precipitation [16]. Local studies have documented occupational heat exposure and residential thermal comfort challenges in specific settings [17,18]. Recruitment for the study was conducted across various institutional and community settings within the city, including comprehensive health centers, university-affiliated clinical and campus settings, public parks, and other public or community locations. The strategic selection of diverse venues facilitated a maximum-variation approach, enabling access to eligible women of varying ages, marital statuses, educational backgrounds, occupational situations, and levels of engagement with formal health and university services. Interviews were conducted in quiet and private conditions, as agreed upon with participants, ensuring the absence of staff, family members, or other non-participants.

### 2.2 Participants and recruitment

Eligible participants were women aged 18–49 years, irrespective of marital status, who had lived in Qom for at least five years, could communicate in Persian, and provided written informed consent. Women were excluded if they self-reported a current psychiatric diagnosis, current use of psychotropic medication, or major acute stressors that could substantially affect the interview content. Eligibility was assessed by self-report without a diagnostic instrument. These criteria were used to delimit the study to a non-clinical community sample; they were not intended to imply that women with psychiatric conditions or acute stressors had fewer valid climate-related experiences. No woman was excluded because of a major acute stressor or solely because of a self-reported psychiatric diagnosis; both exclusions involved current psychotropic medication use. Maximum-variation purposive sampling was used to capture a broad range of experiences rather than to achieve statistical representativeness. The first author recruited participants directly from comprehensive health centers, university-affiliated clinical and campus settings, public parks, and other community locations. Sampling decisions were guided by the eligibility criteria and the need to broaden variation in age, marital status, education, occupational status (student, homemaker, or employed), and recruitment setting as data collection progressed. Eligible women who voluntarily provided informed consent were enrolled, and no snowball sampling or fixed numerical quotas were used. Of the 27 women screened, two were excluded before the interview because of psychiatric medication use. All 25 eligible women participated, and none withdrew from the study. The final sample consequently included women of different ages, marital status, educational background, and occupational status. As appropriate for purposive qualitative sampling, the strategy was not probability-based and did not support estimates of population representativeness.

### 2.3 Data collection

Individual semi-structured interviews were conducted in Persian by the first author, a female university faculty member with a PhD in Reproductive Health, more than ten years of qualitative-research experience, and formal qualitative-methods training. She had no prior relationship with the participants. Before enrolment, participants were informed of the study purpose and the interviewer's institutional and research role.

The semi-structured interview guide covered three connected domains: (1) women's recognition and experiences of climate-related environmental changes; (2) perceived emotional and cognitive challenges; and (3) consequences for everyday and family life. The guide opened with the question, "Please describe your experience of climate change." Further core questions asked, "What concerns and feelings do climate-related changes create for you?", "How do these changes affect your daily and family life?", and "How has your psychological well-being been affected?" Probes elicited concrete examples and explored caregiving, relationships, and future concerns. These domains structured data collection but did not serve as a priori analytical categories. Codes, subcategories, categories, and themes were developed inductively from Persian interview transcripts using conventional content analysis [21]. The complete English interview guide is available in Supplementary File 1.

The first five interviews were treated as pilot interviews. Only the wording was simplified for clarity, and the core domains and prompts remained unchanged, so these interviews were retained in the final analysis. Interviews lasted 35–40 minutes, were audio-recorded with permission, and were not repeated. Formal field notes were not recorded. Each interview was transcribed verbatim, checked against its recording, and preliminary coding preceded the next interview. Participants could add or clarify information at the end of the interview; however, transcripts and findings were not returned for formal participant checking.

Saturation was defined as the point at which consecutive interviews generated no new codes, subcategories, or conceptually distinct insights. It was reached after interview 17 and confirmed by the research team. Eight further interviews produced no substantively new findings, and data collection ended after 25 interviews.

### 2.4 Data analysis

Persian transcripts were analyzed concurrently with data collection using conventional content analysis [21,23]. Meaning units were identified and condensed while preserving their meaning, and codes were assigned. Similar codes were compared and grouped into subcategories and categories and then abstracted into themes.

The first author conducted the primary coding. A second researcher served as the analytical reviewer, examining selected transcripts, meaning units, codes, and the category structure, and participating in discussions of coding decisions. The research team reviewed the analytical structure in team meetings, and disagreements were resolved by consensus. MAXQDA 18 was used for data organization; analytical decisions were made by the researchers.

### 2.5 Translation

Interviews, transcription, coding, and analysis were conducted in Persian, and the full transcripts were not translated for analysis. After the analysis was finalized, the first author translated selected quotations and relevant analytical labels from Persian into English. A bilingual co-author compared these translations with Persian originals, and discrepancies were resolved by consensus to preserve conceptual meaning and context. Formal back-translation was not performed.

### 2.6 Reflexivity and trustworthiness

The interviewer's reproductive-health and qualitative-research background supported contextual understanding but could influence interpretation. Open-ended, non-leading questions and team discussions were used to examine prior assumptions.

Credibility was supported through maximum-variation purposive recruitment across multiple venue types, concurrent data collection and analysis, saturation, primary coding with structured analytical review, and research team review. Dependability was supported by the interview guide and coding records, confirmability by comparison with the original transcripts and consensus review, and transferability by detailed reporting of the recruitment settings, eligibility criteria, participant characteristics, and procedures [24,25]. Formal member checking was not performed.

### 2.7 Ethical considerations

The Research Ethics Committee of Qom University of Medical Sciences approved the study on 15 April 2024 (approval ID: IR.MUQ.REC.1403.012). Written informed consent was obtained before the interviews. Participants were informed about voluntary participation, confidentiality, audio recording, and their right to withdraw without penalties or loss of service. Numerical codes replaced identifiers, and recordings, transcripts, and analytical records were stored in password-protected files that were accessible only to the research team.

## 3 Results

Twenty-five women participated, with a mean age of 34.4 years (SD 9.4; range, 18–45 years); their characteristics are shown in Table 1. The analysis generated four main themes, nine categories, and 17 subcategories (Table 2): (1) Living with a disrupted and increasingly insecure environment; (2) consequences of climate change in everyday life; (3) climate-related psychological distress; and (4) social consequences of climate change for mental health. Together, these themes show how perceived environmental changes entered bodily experience, caregiving, household economics, emotional responses, cognitive preoccupation, moral reasoning, and social life. To contextualize the recurrence of findings, n (%) indicates the number and proportion of the 25 participants whose accounts contributed at least one meaning unit to a subcategory; each participant was counted once per subcategory. These descriptive frequencies indicate recurrence within the qualitative sample and should not be interpreted as prevalence estimates. Additional illustrative quotations by theme, category, and subcategory are presented in Table S1.

**Table 1:** Demographic characteristics of participants.

Participant no.	Age (years)	Marital status	Education	Employment/occupational status
1	24	Single	Bachelor's degree	Employed
2	36	Married	Bachelor's degree	Homemaker
3	18	Single	High school	Student
4	39	Married	Bachelor's degree	Homemaker
5	43	Married	Bachelor's degree	Employed
6	42	Married	Bachelor's degree	Employed
7	43	Single	Bachelor's degree	Employed
8	37	Married	Master’s degree	Employed
9	45	Married	Bachelor's degree	Employed
10	40	Married	Bachelor's degree	Homemaker
11	21	Single	Undergraduate student	Student
12	23	Single	Undergraduate student	Student
13	22	Single	Undergraduate student	Student
14	22	Single	Undergraduate student	Student
15	40	Married	High school diploma	Homemaker
16	41	Married	High school	Homemaker
17	39	Married	High school diploma	Homemaker
18	45	Married	High school diploma	Homemaker
19	39	Married	Bachelor's degree	Employed
20	44	Married	Bachelor's degree	Homemaker
21	40	Married	Bachelor's degree	Homemaker
22	22	Married	Bachelor's degree	Homemaker
23	20	Married	High school diploma	Homemaker
24	35	Married	Master’s degree	Employed
25	39	Married	Bachelor's degree	Homemaker

**Table 2:** Themes, categories, subcategories, and participant-level recurrence derived from women's experiences of climate change and mental health.

Main theme	Category	Subcategory	Participants, n (%)
3.1. Living with a disrupted and increasingly insecure environment	3.1.1. Perceived environmental disruption and insecurity	3.1.1.1. Disrupted climatic order and intensifying heat	20 (80.0)
		3.1.1.2. Drought and water insecurity as everyday reality	19 (76.0)
3.2. Consequences of climate change in everyday life	3.2.1. Bodily and economic consequences	3.2.1.1. Embodied consequences of heat, saline water, and polluted air	17 (68.0)
		3.2.1.2. Household costs and livelihood pressure	15 (60.0)
	3.2.2. Environmental and household consequences	3.2.2.1. Accumulated environmental pressure: heat, dust, and air pollution	17 (68.0)
		3.2.2.2. Food quality and agricultural disruption	12 (48.0)
		3.2.2.3. Climate-related residential preferences	4 (16.0)
3.3. Climate-related psychological distress	3.3.1. Emotional manifestations of psychological distress	3.3.1.1. Anticipatory fear of worsening climatic conditions	17 (68.0)
		3.3.1.2. Heat-related mood changes and reduced social tolerance	10 (40.0)
		3.3.1.3. Place-based grief and reduced psychological restoration	13 (52.0)
	3.3.2. Cognitive manifestations of psychological distress	3.3.2.1. Reduced precipitation as a warning of future hardship	14 (56.0)
		3.3.2.2. Children’s current health and caregiving responsibility	6 (24.0)
		3.3.2.3. Future insecurity and children’s liveability	5 (20.0)
	3.3.3. Moral manifestations of psychological distress	3.3.3.1. Moral self-monitoring during household water use	8 (32.0)
3.4. Social consequences of climate change for mental health	3.4.1. Restricted restorative relationships and family activities	3.4.1.1. Disrupted restorative social and family activities	7 (28.0)
	3.4.2. Climate-related work disruption and livelihood insecurity	3.4.2.1. Work routines, agricultural loss, and mental health	15 (60.0)
	3.4.3. Resource scarcity and household tension	3.4.3.1. Resource monitoring and family conflict	5 (20.0)

Note: n (%) represents the number and percentage of the 25 participants who contributed at least one meaning unit to the corresponding subcategory. Each participant was counted once per subcategory; percentages are descriptive and are not prevalence estimates.

### 3.1 Living with a disrupted and increasingly insecure environment

#### 3.1.1 Perceived environmental disruption and insecurity

##### 3.1.1.1 Disrupted climatic order and intensifying heat

Twenty participants (80.0%) described this subcategory. Women contrasted past and present environmental conditions and interpreted reduced rainfall, declining groundwater, seasonal irregularity, and intensified heat as evidence that the familiar climatic order had become less stable and predictable.

“I remember that, in the past, everything had its own order. However, rainfall has decreased significantly, and groundwater levels have fallen. These were changes I did not notice at all in the past.”

(Participant 2, 36 years)

##### 3.1.1.2 Drought and water insecurity as everyday reality

Drought and water insecurity were described by 19 participants (76.0%). Reduced rainfall, prolonged dry periods, declining water availability, and possible rationing were described not as temporary events but as persistent conditions that weakened the participants’ sense of environmental security.

“In recent years, the fear of water shortages has increased significantly. Rainfall has decreased, and prolonged drought has become part of the reality of our lives.”

(Participant 25, 39 years)

### 3.2 Consequences of climate change in everyday life

The women described the daily consequences of heat, saline water, air pollution, dust, drought, and reduced rainfall. These conditions shape bodily discomfort, household and health-related costs, food quality, agricultural livelihoods, work routines, and residential preferences.

#### 3.2.1 Bodily and economic consequences

##### 3.2.1.1 Embodied consequences of heat, saline water, and polluted air

Embodied consequences were described by 17 participants (68.0%). Heat and saline water were associated with skin and hair problems, whereas polluted air, dust, and suspended particles were linked to respiratory symptoms, allergies, sleep disturbance, and concern for family health.

“The heat and the salinity of the water in Qom have caused hair loss and dry skin for me.”

(Participant 1, 24 years)

##### 3.2.1.2 Household costs and livelihood pressure

Household costs and livelihood pressures were described by 15 participants (60.0%). Participants linked climatic conditions to health-related expenses, damage to household equipment, reduced agricultural productivity, income insecurity, and concerns shared across families.

“My uncles and grandfather are farmers. Last year, rainfall was low, and there was a drought, which worried them. Their walnut trees did not produce a good crop, and their yields decreased. This issue was repeatedly discussed in family gatherings.”

(Participant 3, 18 years)

#### 3.2.2 Environmental and household consequences

##### 3.2.2.1 Accumulated environmental pressure: heat, dust, and air pollution

Accumulated environmental pressure was described by 17 participants (68.0%). Heat was rarely experienced in isolation and was described alongside dust, fine particulate matter, polluted air, and the need to keep doors and windows closed, making everyday life in Qom more difficult.

“Qom has always been a hot city, but in recent years, in addition to heat, we have also been dealing with dust and air pollution, and this is truly a serious issue.”

(Participant 20, 44 years)

##### 3.2.2.2 Food quality and agricultural disruption

Food quality and agricultural disruptions were described by 12 (48.0%) participants. Irregular seasons, drought, water scarcity, and frost damage were perceived as reducing crop yields and food quality, thereby bringing environmental changes to household food consumption and well-being.

“Drought and water scarcity greatly affect the quality of food and fruit. When we buy fruit, the quality of many items is low because there is not enough water to grow them. In the end, these conditions make our lives more unpleasant.”

(Participant 17, 39 years)

##### 3.2.2.3 Climate-related residential preferences

Climate-related residential preferences were described by four participants (16.0%). Qom’s hot and dry climate shaped how these women imagined future living arrangements, with cooler and greener locations perceived as offering greater comfort and liveability.

“Many people leave Qom. For example, if I were to get married, I would prefer not to live in Qom and would move to a city with a cooler climate.”

(Participant 14, 22 years)

### 3.3 Climate-related psychological distress

Psychological distress was used descriptively, not diagnostically, as an umbrella for interrelated emotional, cognitive, and moral manifestations. Emotional manifestations included stress, fear, worry, grief, irritability, and reduced vitality; cognitive manifestations included recurrent anticipation, future-oriented preoccupation, and uncertainty about resources and children’s liveability; and moral manifestations were limited to self-monitoring of household water use, including self-questioning about possible waste and, in some accounts, guilt or worry that personal water use might contribute to scarcity. Categories reflected the dominant meaning of participants’ accounts despite overlap.

#### 3.3.1 Emotional manifestations of psychological distress

##### 3.3.1.1 Anticipatory fear of worsening climatic conditions

Seventeen participants (68.0%) described fear and anxiety about worsening drought, water scarcity, reduced rainfall, pollution, rising temperatures, and future liveability.

“In recent years, I have constantly been afraid of drought, water scarcity, and reduced rainfall. The fact that the situation gets worse every year makes me more worried and more nervous.”

(Participant 2, 36 years)

##### 3.3.1.2 Heat-related mood changes and reduced social tolerance

Ten participants (40.0%) linked high temperatures with irritability, restlessness, fatigue, reduced patience, and difficulty maintaining expected social conduct in public settings.

“Rising temperatures reduce people’s tolerance and resilience in society. During hot seasons, especially when temperatures rise, people are less patient, particularly in public spaces. They have less tolerance and cannot observe social norms as expected.”

(Participant 23, 20 years)

##### 3.3.1.3 Place-based grief and reduced psychological restoration

Thirteen participants (52.0%) described place-based grief arising when dry rivers, declining greenery, and reduced contact with nature transformed familiar landscapes into reminders of loss, evoking regret, sadness, anger, and reduced vitality.

“On my daily route to work, I used to feel calm because of the river. Now, when I pass by the dry riverbed, I feel regret and anger. The absence of water has made me feel discouraged and sad.”

(Participant 8, 37 years)

#### 3.3.2 Cognitive manifestations of psychological distress

##### 3.3.2.1 Reduced precipitation as a warning of future hardship

Fourteen participants (56.0%) interpreted reduced precipitation primarily as a warning of future hardship. Despite its emotional meaning, the dominant pattern was cognitive appraisal of likely summer heat, water shortages, and electricity rationing.

*“It rains much less now than in the past. Before, when it rained, it gave me a great sense of calmness. Perhaps it is subconscious, but now it feels as though we have lost something, and a kind of worry has taken over me.”*

*(Participant 6, 42 years)*

##### 3.3.2.2 Children’s current health and caregiving responsibility

Six participants (24.0%) described current preoccupation with children’s exposure to polluted air and heat and its implications for school attendance, illness, caregiving decisions, and outdoor play.

“In winter, when you want to send children to school in dry and polluted air, you become worried; it is truly frightening.”

(Participant 17, 39 years)

##### 3.3.2.3 Future insecurity and children’s liveability

Five participants (20.0%) described future-oriented preoccupation with resource availability, safe living conditions, and whether Qom would remain liveable for their children and future generations.

“I am worried about water scarcity, air pollution, and lack of resources in the future. I am worried about my children’s future. Sometimes I feel that the world is coming to an end and that we are living in extra time.”

(Participant 2, 36 years)

#### 3.3.3 Moral manifestations of psychological distress

##### 3.3.3.1 Moral self-monitoring during household water use

Eight participants (32.0%) described self-monitoring during household water use. Their accounts involved self-questioning about possible overuse or waste during washing, cleaning, bathing, toothbrushing, and ablution; some explicitly described guilt or worry that their water use might waste water or contribute to scarcity. Unlike anticipatory fear, the defining feature was moral appraisal of immediate conduct.

“When I use water, I always feel guilty and worried that I might be wasting it.”

(Participant 7, 43 years)

### 3.4 Social consequences of climate change for mental health

Participants described how heat, water scarcity, dryness, and air pollution extended into social relationships, family activities, work routines, livelihoods, and household interactions.

#### 3.4.1 Restricted restorative relationships and family activities

##### 3.4.1.1 Disrupted restorative social and family activities

Disruption of restorative social and family activities was described by seven participants (28.0%). Unfavourable weather, dryness, water scarcity, and air pollution reduce opportunities to go outdoors, exercise, play, visit natural settings, and spend restorative time with relatives.

“Before, when I woke up in the morning and the weather was good, I would call my mother and sister so that we could go out together. However, now, because it does not rain and the air is polluted, all our plans are disrupted. This makes me sad and anxious.”

(Participant 6, 42 years)

#### 3.4.2 Climate-related work disruption and livelihood insecurity

##### 3.4.2.1 Work routines, agricultural loss, and mental health

Work disruption and livelihood insecurity were reported by 15 (60.0%) participants. Extreme heat shifted activities toward cooler hours, while drought and water scarcity restricted agricultural work, reduced income, and generated anxiety about losing the remaining livelihood opportunities.

“Due to water scarcity and drought, they no longer have sufficient water to work on the farmland, and they cannot work as they did before. Gradually, this situation affected their mental health and caused distress and irritability. They constantly feel anxious about losing their work.”

(Participant 6, 42 years)

#### 3.4.3 Resource scarcity and household tension

##### 3.4.3.1 Resource monitoring and family conflict

Resource monitoring and family conflict were described by five participants (20.0%). Repeated reminders about water, electricity, heating, and cooling transform resource conservation from a practical household task to a source of emotional pressure and interpersonal tension.

“My mother constantly repeats that we should use less electricity, turn off the cooler sooner, and use less water. These repeated reminders sometimes make my brother very upset and lead to tension and conflict between them.”

(Participant 12, 23 years)

## 4 Discussion

This study aimed to explore how reproductive-aged women in Qom perceived and experienced climate-related psychological distress in the context of chronic environmental change. Rather than representing four separate sets of concerns, the findings suggest a provisional situated cumulative pathway in which perceived environmental changes are connected with bodily discomfort and material pressures, while their significance is shaped by household routines, caregiving, resource management, place attachment, and uncertainty about the future. Psychological distress was used descriptively rather than diagnostically and was organized into emotional, cognitive, and moral manifestations according to the dominant meaning of participants’ accounts, while acknowledging overlap. It was therefore interpreted neither as a response to a single hazard nor as an isolated mental state, but as arising within the interaction of environmental, economic, caregiving, relational, and place-based pressures. This pathway is offered as an interpretive synthesis of participants’ accounts and not as a tested causal model.

The cumulative characteristics of this pathway are important in Qom. Participants often described heat alongside dust and polluted air, while water-related concerns, including scarcity and salinity, appeared across bodily, household, and future-oriented accounts. Across participants’ accounts, these perceived stressors were linked to physical discomfort; keeping windows closed; limiting outdoor activity; higher cooling and health-related expenses; monitoring water and electricity use; and worry about food, work, and children. These overlapping demands may reduce opportunities for psychological restoration while increasing the practical, cognitive, and emotional work required to maintain ordinary family life. Thus, the significance of chronic climate-related pressure may lie partly in its capacity to simultaneously affect several domains of daily life. This interpretation is consistent with frameworks describing direct, indirect, and overarching pathways from climate change to mental health, and with evidence that slow-onset environmental change can compound existing social and economic stressors [3,20,26].

These findings add a locally situated account of how these pathways were experienced in a dry Iranian city. In a recent qualitative study of women and men of reproductive age in Qom, participants similarly connected extreme heat, dust, air pollution, resource scarcity, indoor confinement, economic insecurity, parental responsibility, and concern for children’s futures with reproductive decision-making [10]. The convergence between these two studies supports the contextual relevance of these concerns in Qom. However, because the earlier study included both women and men and did not stratify all themes by sex, neither study can establish that the pressures identified here are uniquely female [10]. Qualitative research from Bangladesh has likewise shown that environmental and socioeconomic pressures can accumulate through household strategies, livelihood disruptions, and care responsibilities [20]. Among young adults in India, fear, eco-guilt, eco-grief, environmental vigilance, and calls for structural action have also been reported [27]. Together, these studies suggest that climate-related psychological responses in the Asian settings examined were embedded in local material and social conditions rather than experienced only as abstract concerns about a global threat [10,20,27].

Within this broader context, caregiving may amplify the salience of perceived environmental pressures and the responsibilities attached to them. Participants did not simply worry about climate change in general; they described having to decide whether children could attend school or play outdoors, respond to illnesses or polluted air, maintain cooling, manage household costs, conserve water, and anticipate whether essential resources would remain available. These caregiving concerns were classified within the cognitive dimension because their dominant content involved risk appraisal, planning, and decision-making, although fear and worry often accompanied those processes. These recurring tasks may make environmental change more immediate because the person responsible for care repeatedly encounters practical consequences and must make decisions for others as well as herself. Comparable role-based mechanisms were reported in Bangladesh, where caregiving, household labor, food insecurity, and livelihood pressures shaped women’s psychological experiences of climate change [20]. Broader reviews also describe gendered socioeconomic stressors and associated mental health vulnerability among women [8,9]. However, the present study can only explain how women experienced these responsibilities; it cannot determine whether the burdens were unique to women or greater than those experienced by men. The gendered interpretation should therefore be understood as context-specific and role-based, not as evidence of inherent female vulnerability.

Participants’ narratives further suggested that climate-related psychological distress involved analytically distinguishable but overlapping emotional, cognitive, and moral manifestations. Emotional manifestations included anticipatory fear, heat-related irritability, and place-based grief. Cognitive manifestations involved appraisal of reduced precipitation as a warning, ongoing preoccupation with children’s current exposures, and uncertainty about future resources and liveability. Moral manifestations were limited to household water-use self-monitoring: participants questioned possible waste, and some described guilt or worry that their use might waste water or contribute to scarcity. These patterns overlap: anticipation can involve both thought and fear, while water-use monitoring is cognitive in form but moral in its evaluative focus on one’s own conduct and possible waste. Relational consequences were evident in reduced social tolerance and conflict over water, electricity, and cooling. Similar findings from India include fear, eco-guilt, eco-grief, and environmental vigilance [27]. These responses should not automatically be interpreted as psychiatric symptoms; anxiety, worry, and grief can be understandable and sometimes constructive responses to environmental threats [7]. Because participants linked these responses with daily functioning, family relationships, and reduced restorative activity, they are relevant to public health and may warrant attention in primary care settings [2]. A non-pathologizing response should recognize their meaning while offering support when distress becomes persistent, overwhelming, or functionally impairing [2,7].

The concept of solastalgia provides a particularly useful bridge across environmental, psychological, and social findings. Dry riverbeds, loss of greenery, reduced rainfall, and disrupted familiar outdoor routines represented more than dissatisfaction with environmental quality. Participants remained in the same city, yet the environmental features through which they experienced calm, continuity, and familiarity had diminished. This resembles solastalgia: distress arising when a person’s home environment changes while the person remains connected to it [28]. Analytically, reduced precipitation was classified primarily as cognitive when participants appraised it as a warning of future hardship, whereas grief was classified as emotional when attached to the loss of specific familiar landscapes and restorative experiences. In this study, the apparent loss of restorative landscapes may have also narrowed opportunities for going outdoors, exercising, visiting natural settings, and spending time with relatives. It may therefore affect not only individual mood but also the everyday practices through which attachment to place and social connection are maintained. Similarly, research on climate change, sense of place, and ecological grief indicates that changes in land and valued landscapes can affect mental and emotional well-being, cultural practices, livelihoods, and sense of place [6,29]. The present findings do not demonstrate a loss of social cohesion, but they suggest a plausible process through which the degradation of familiar places may weaken some of the shared routines that sustain it.

Taken together, the findings elaborate on how climate-related psychological distress is experienced in this setting. The proposed situated cumulative pathway connects (1) perceived chronic and overlapping environmental stressors; (2) embodied and material consequences; (3) household, caregiving, and livelihood demands; and (4) overlapping emotional, cognitive, moral, and relational manifestations of distress. These components may reinforce each other. For example, participants linked heat and polluted air with restricted outdoor activity and reduced restoration, while also describing concerns about children’s health and household tension around water, electricity, and cooling. This interpretation suggests that participants’ distress may not be adequately understood through a single exposure or emotion. It also supports the consideration of responses that address both psychosocial needs and the local conditions through which distress is experienced.

The findings suggest priorities for local co-design and evaluation rather than established interventions. Such responses should distinguish understandable climate-related emotions and cognitions from clinically impairing distress, address water-use-related moral concern without individualizing responsibility for structural scarcity, and link psychosocial support with practical adaptation. Potential entry points include non-diagnostic inquiry and referral in comprehensive health centres, outreach through university and community services, practical heat and air-quality guidance, and measures that preserve access to safe, shaded, and restorative social spaces. These proposals align broadly with WHO calls for multisectoral and community-based approaches; however, their feasibility, acceptability, and effectiveness should be tested with women, families, service providers, and local authorities [2]. Because coping strategies were outside the scope of the present analysis, future research should ask women which individual, family, social, spiritual or religious, and service-based strategies, if any, they consider helpful and should use participatory methods to translate those insights into locally acceptable support. Evaluation of existing or newly piloted measures across Qom’s health center, university, and community channels could examine feasibility, acceptability, unintended effects, and changes in distress and daily functioning. Comparative studies, including men and people of different ages and socioeconomic positions, would help distinguish widely shared climate-related pressures from those shaped by gender roles. Mixed-methods and longitudinal designs that combine lived experience with objective heat, air quality, water, and service use data could also examine the proposed cumulative pathway without making causal claims from qualitative accounts alone.

### 4.1 Limitations

This study had several limitations. The findings reflect participants’ perceptions of environmental change. In the absence of objective environmental or clinical measurements, reported experiences cannot be attributed to specific exposures or interpreted causally. The organization of emotional, cognitive, and moral manifestations reflects the dominant meaning of participants’ accounts; these processes overlapped and should not be treated as mutually exclusive psychological constructs. Because only women were interviewed and no comparison group was included, the study could not determine whether these burdens were unique to women or greater than those experienced by men. Maximum-variation purposive sampling broadened the range of accounts but was not population-based and may have underrepresented women outside the recruitment settings and socioeconomically marginalized groups, limiting transferability to rural Qom and other geographical or sociocultural contexts. Excluding women who reported a current psychiatric diagnosis, psychotropic medication use, or a major acute stressor may have reduced the representation of those with greater pre-existing vulnerability; without diagnostic assessment, some misclassification is possible. Social desirability and recruitment context may also have influenced sensitive accounts. Finally, despite bilingual checking and consensus-based resolution of discrepancies, translation from Persian to English may have reduced some cultural and emotional nuances.

## 5 Conclusion

Women of reproductive age in Qom described climate-related psychological distress as a daily, situated experience embedded in caregiving, household management, work, family relationships, and place attachment. Emotional manifestations included fear, irritability, and place-based grief; cognitive manifestations included appraisal of worsening conditions, caregiving preoccupation, and uncertainty about future resources and liveability; and moral manifestations involved household water-use self-monitoring, including self-questioning about waste and, in some accounts, guilt or worry about wasting water. These overlapping responses were linked to heat, water scarcity, salinity, air pollution, dust, drought, and environmental degradation within a provisional, non-causal cumulative pathway. They should be acknowledged without automatic pathologization. The findings support co-designing and evaluating locally appropriate practical and psychosocial responses through primary care, university, and community services in Qom.

## Ethics approval and consent to participate

The Research Ethics Committee of Qom University of Medical Sciences approved the study on 15 April 2024 (approval ID: IR.MUQ.REC.1403.012).

## Declaration of generative AI and AI-assisted technologies in the manuscript preparation process

During the preparation of this work, the authors used ChatGPT (OpenAI) to support language editing. After using this tool, the authors reviewed and edited the content as needed and take full responsibility for the content of the published article.

## Funding

This work was supported by Qom University of Medical Sciences. The funder had no role in study design, data collection, analysis, interpretation, manuscript preparation, or the decision to submit the article.

## CRediT authorship contribution statement

**Zahra Alipour:** Writing – review & editing, Writing – original draft, Visualization, Validation, Supervision, Software, Resources, Project administration, Methodology, Investigation, Funding acquisition, Formal analysis, Data curation, Conceptualization. **Maryam Ghaedrahmati:** Investigation, Funding acquisition, Formal analysis, Data curation, Conceptualization. **Faride Yousefizadeh:** Writing – original draft, Visualization, Validation, Resources.

## Declaration of competing interest

The authors declare that they have no known competing financial interests or personal relationships that could have appeared to influence the work reported in this paper.

## Data Availability

The de-identified interview transcripts are not publicly available because they contain sensitive qualitative data that could compromise participant confidentiality and because public sharing was not included in the consent process. De-identified excerpts supporting the findings are included in the article and Supplementary Table S1. Requests for additional de-identified information will be considered by the corresponding author, subject to ethics committee and institutional approval and to the limits of participant consent.

## Footnotes

Supplementary material associated with this article can be found, in the online version, at doi:10.1016/j.joclim.2026.100760.

## References

[1] Intergovernmental Panel on Climate Change. Climate change 2022: impacts, adaptation and vulnerability. Contribution of Working Group II to the Sixth Assessment Report of the Intergovernmental Panel on Climate Change. Pörtner H.O., Roberts D.C., Tignor M., Poloczanska E.S., Mintenbeck K., Alegría A., et al., editors. Cambridge (UK) and New York (NY): Cambridge University Press; 2022. 10.1017/9781009325844.

[2] World Health Organization (2022).

[3] Burrows K., Denckla C.A., Hahn J., Schiff J.E., Okuzono S.S., Randriamady H. (2024). A systematic review of the effects of chronic, slow-onset climate change on mental health. Nat Ment Health.

[4] Clayton S. (2021). Climate change and mental health. Curr Environ Health Rep.

[5] Clayton S., Karazsia B.T. (2020). Development and validation of a measure of climate change anxiety. J Environ Psychol.

[6] Cunsolo Willox A., Stephenson E., Allen J., Bourque F., Drossos A., Elgarøy S. (2015). Examining relationships between climate change and mental health in the Circumpolar North. Reg Environ Change.

[7] Ojala M., Cunsolo A., Ogunbode C.A., Middleton J. (2021). Anxiety, worry, and grief in a time of environmental and climate crisis: a narrative review. Annu Rev Environ Resour.

[8] Anjum G., Aziz M. (2025). Climate change and gendered vulnerability: a systematic review of women's health. Womens Health (Lond).

[9] Rothschild J., Haase E. (2023). Women's mental health and climate change part II: socioeconomic stresses of climate change and eco-anxiety for women and their children. Int J Gynaecol Obstet.

[10] Alipour Z., Ghaedrahmati M., Taghvi S. (2025). Navigating parenthood in a climate change era: determinants of childbearing intentions in Iran. Sci Rep.

[11] Dillarstone H., Brown L.J., Flores E.C. (2023). Climate change, mental health, and reproductive decision-making: a systematic review. PLOS Clim.

[12] Alipour Z., Eskandari N., Sangi S., Gafarbegloo E., Ahmari Tehran H., Aghvam Karbasi Z. (2019). Health need assessment in female adolescents, in Qom: a cross-sectional study. J Educ Health Promot.

[13] Karimi V., Karami E., Keshavarz M. (2018). Climate change and agriculture: impacts and adaptive responses in Iran. J Integr Agric.

[14] Rahimi J., Malekian A., Khalili A. (2019). Climate change impacts in Iran: assessing our current knowledge. Theor Appl Climatol.

[15] Asadi-RahimBeygi N., Mofidi A., Zarrin A., Dadashi-Roudbari A. (2025). Near-term climate extremes in Iran based on compound hazards analysis. Sci Rep.

[16] Heydari M.M., Aghamajidi R., Beygipoor G., Heydari M. (2014). Comparison and evaluation of 38 equations for estimating reference evapotranspiration in an arid region. Fresenius Environ Bull.

[17] Hajizadeh R., Golbabaei F., Monazzam M.R., Farhang-Dehghan S., Ezadi-Navan E. (2014). Productivity loss from occupational exposure to heat stress: a case study in brick workshops/Qom-Iran. Int J Occup Hyg.

[18] Taher Tolou Del M.S., Bayat S., Zojaji N. (2022). The effect of building plan form on thermal comfort in the traditional residential patterns of the hot and dry climate of Qom. Herit Sci.

[19] Samani S. (2021). Assessment of groundwater sustainability and management plan formulations through the integration of hydrogeological, environmental, social, economic and policy indices. Groundw Sustain Dev.

[20] Goudet J.M., Binte Arif F., Owais H., Uddin Ahmed H., Ridde V. (2024). Climate change and women's mental health in two vulnerable communities of Bangladesh: an ethnographic study. PLOS Glob Public Health.

[21] Bengtsson M. (2016). How to plan and perform a qualitative study using content analysis. NursPlus Open.

[22] Tong A., Sainsbury P., Craig J. (2007). Consolidated criteria for reporting qualitative research (COREQ): a 32-item checklist for interviews and focus groups. Int J Qual Health Care.

[23] Graneheim U.H., Lundman B. (2004). Qualitative content analysis in nursing research: concepts, procedures and measures to achieve trustworthiness. Nurse Educ Today.

[24] Lincoln Y.S., Guba E.G. (1986). But is it rigorous? Trustworthiness and authenticity in naturalistic evaluation. New Dir Program Eval.

[25] Ahmed S.K. (2024). The pillars of trustworthiness in qualitative research. J Med Surg Public Health.

[26] Hayes K., Blashki G., Wiseman J., Burke S., Reifels L. (2018). Climate change and mental health: risks, impacts and priority actions. Int J Ment Health Syst.

[27] Mannan P.M., Basu S. (2026). Understanding eco-anxiety and lived experiences of young adults in India about climate change: a qualitative study. J Clim Chang Health.

[28] Albrecht G., Sartore G.M., Connor L., Higginbotham N., Freeman S., Kelly B. (2007). Solastalgia: the distress caused by environmental change. Australas Psychiatry.

[29] Cunsolo A., Ellis N.R. (2018). Ecological grief as a mental health response to climate change-related loss. Nat Clim Change.

