# Supplementary material for: Climate-related psychological distress among reproductive-age women: A qualitative content analysis
Source: J Clim Chang Health. 2026 Oct 28;32:100760. doi: 10.1016/j.joclim.2026.100760 (PMC13742310; doi:10.1016/j.joclim.2026.100760)
Supplement: Supplementary file 1 [file mmc1.docx]

**SUPPLEMENTARY FILE 1**

**Semi-structured Interview Guide**

*Climate-related psychological distress among reproductive-age women: A qualitative content analysis*

**Purpose**

To explore reproductive-age women’s experiences of climate change in Qom, Iran, including their worries and feelings, perceived effects on everyday life and mental well-being, and related family and social consequences.

**Administration and opening**

The guide was administered in Persian during individual face-to-face interviews. The primary questions were open-ended and could be adapted to the interview flow. Broad questions were asked before optional probes, which were used selectively to clarify or deepen the participant’s own account rather than as a checklist. After explaining the study purpose, confidentiality, voluntary participation, audio recording, and the right to withdraw, the interviewer invited the participant to describe her experiences freely. Interviews lasted approximately 35-40 min and were audio-recorded after written informed consent.

**Primary interview questions and optional probes**

1. Please describe your experience of climate change or changes in weather and climate and, if relevant, how this experience relates to your mental well-being.

2. Please describe the worries, concerns, and feelings you experience in relation to climate change or its consequences.

3. How, if at all, have climate-related changes affected your everyday life?

4. How, if at all, has your mental well-being been affected by climate change or its consequences?

**General clarification prompts**

- Could you explain this further?
- Could you provide a specific example?
- When did you first notice this experience or change?
- What happened next, and how did this affect you?

**Closing question**

Is there anything else about climate change and its effects on your psychological well-being or everyday life that you would like to add?

**Pilot testing and translation note**

The first five interviews served as pilot interviews. The wording of questions relating to climate change was simplified for clarity, but no substantive change was made to the guide; these interviews were retained in the final dataset. The English guide presented here is a conceptually reviewed translation of the final Persian guide. The questions guided data collection but did not predetermine the inductively developed codes, categories, or themes.
