# Supplementary material for: Climate-related psychological distress among reproductive-age women: A qualitative content analysis
Source: J Clim Chang Health. 2026 Oct 28;32:100760. doi: 10.1016/j.joclim.2026.100760 (PMC13742310; doi:10.1016/j.joclim.2026.100760)
Supplement: Supplementary file 2 [file mmc2.docx]

**SUPPLEMENTARY FILE 2**

**Completed COREQ Checklist**

Climate-related psychological distress among reproductive-age women: A qualitative content analysis

Reporting framework: Consolidated Criteria for Reporting Qualitative Research (COREQ), 32-item checklist for interviews and focus groups. Locations correspond to the final marked manuscript.

| **No.** | **COREQ item** | **Study-specific response** | **Manuscript location** |
| --- | --- | --- | --- |
| **Domain 1: Research team and reflexivity** | | | |
| 1 | Interviewer/facilitator | Interviews were conducted by the first author. | Marked manuscript: Methods, p. 4, lines 17-21. |
| 2 | Credentials | The interviewer held a PhD in Reproductive Health. | Marked manuscript: Methods, p. 4, lines 17-19. |
| 3 | Occupation | She was a university faculty member. | Marked manuscript: Methods, p. 4, lines 17-19. |
| 4 | Gender | The interviewer was female. | Marked manuscript: Methods, p. 4, lines 17-19. |
| 5 | Experience and training | She had more than ten years of qualitative-research experience and formal doctoral-level and workshop-based qualitative-methods training. | Marked manuscript: Methods, p. 4, lines 17-19. |
| 6 | Relationship established | The interviewer had no prior personal or professional relationship with participants. | Marked manuscript: Methods, p. 4, lines 19-20. |
| 7 | Participant knowledge of the interviewer | Before enrolment, participants were informed about the study purpose and the interviewer's institutional and research role. | Marked manuscript: Methods, p. 4, lines 20-21. |
| 8 | Interviewer characteristics | Her reproductive-health and qualitative-research background and their possible influence on interpretation were addressed through open-ended interviewing and team discussions. | Marked manuscript: Methods, p. 5, lines 26-29. |
| **Domain 2: Study design** | | | |
| 9 | Methodological orientation and theory | Conventional qualitative content analysis informed by Graneheim and Lundman and Bengtsson was used. | Marked manuscript: Methods, p. 3, lines 15-20 and p. 5, lines 8-18. |
| 10 | Sampling | Maximum-variation purposive sampling was conducted across multiple institutional and community settings. Sampling deliberately sought variation in age, marital status, education, occupational status (student, homemaker, or employed), and recruitment setting. No fixed quota, probability-based selection, or statistical representativeness is claimed. | Marked manuscript: Methods, p. 3, lines 21-30 and p. 4, lines 3-15. |
| 11 | Method of approach | The first author directly approached potentially eligible women at comprehensive health-centre, university-affiliated clinical or campus, public-park, and other community settings in Qom. Women who met the eligibility criteria and voluntarily consented were enrolled in accordance with the maximum-variation strategy. No snowball sampling was used. | Marked manuscript: Methods, p. 4, lines 3-10. |
| 12 | Sample size | Twenty-five women participated. | Marked manuscript: Methods, p. 4, lines 10-13; Results, p. 6, lines 8-10. |
| 13 | Non-participation | Of 27 women screened, two were ineligible because of psychiatric medication use. No eligible woman declined or withdrew. | Marked manuscript: Methods, p. 4, lines 10-12. |
| 14 | Setting of data collection | Recruitment occurred across health-centre, university, park, and other community venues. Interviews were conducted under quiet, private conditions agreed with participants. | Marked manuscript: Methods, p. 3, lines 21-30 and p. 4, lines 3-6. |
| 15 | Presence of non-participants | No staff member, family member, or other non-participant was present during interviews. | Marked manuscript: Methods, p. 3, lines 28-30. |
| 16 | Description of sample | Age, marital status, educational level, and occupation are reported. | Marked manuscript: Results, p. 6, lines 8-10; Table 1, p. 19. |
| 17 | Interview guide | A semi-structured guide was used. The first five interviews served as pilots; only the wording of climate-change questions was simplified. | Marked manuscript: Methods, p. 4, lines 22-36; Supplementary File 1. |
| 18 | Repeat interviews | No interview was repeated. | Marked manuscript: Methods, p. 4, line 37 to p. 5, line 1. |
| 19 | Audio/visual recording | All interviews were digitally audio-recorded with permission. | Marked manuscript: Methods, p. 4, lines 37-38. |
| 20 | Field notes | No formal field notes were taken. | Marked manuscript: Methods, p. 4, line 38. |
| 21 | Duration | Interviews lasted approximately 35-40 min. | Marked manuscript: Methods, p. 4, line 37. |
| 22 | Data saturation | Saturation was reached after interview 17; eight further interviews confirmed that no substantively new findings emerged. | Marked manuscript: Methods, p. 5, lines 4-7. |
| 23 | Transcripts returned | Transcripts were not returned to participants for correction or comment. | Marked manuscript: Methods, p. 5, lines 1-3. |
| **Domain 3: Analysis and findings** | | | |
| 24 | Number of data coders | One primary coder (the first author) conducted coding. A second researcher served as an analytical reviewer, examining selected transcripts, meaning units, codes, and category development; the research team reviewed the analytical structure and resolved disagreements by consensus. | Marked manuscript: Methods, p. 5, lines 8-18 and 26-36. |
| 25 | Description of coding tree | Meaning units were condensed and coded; similar codes formed 17 subcategories and nine categories, which were abstracted into four themes. | Marked manuscript: Methods, p. 5, lines 9-16; Table 2, pp. 19-20. |
| 26 | Derivation of themes | Codes, categories, and themes were derived inductively from the Persian interview data. | Marked manuscript: Methods, p. 4, lines 29-33 and p. 5, lines 8-16. |
| 27 | Software | MAXQDA version 18 was used for data organisation and retrieval. | Marked manuscript: Methods, p. 5, lines 17-18. |
| 28 | Participant checking | No formal participant checking of transcripts or findings was undertaken. | Marked manuscript: Methods, p. 5, lines 1-3 and 26-36. |
| 29 | Quotations presented | Illustrative quotations are identified by participant code and age; additional quotations are provided in Supplementary Table S1. | Marked manuscript: Results, p. 6, line 23 to p. 13, line 21; Supplementary Table S1. |
| 30 | Data and findings consistent | Themes and subcategories are supported by participant quotations and participant-level recurrence data. | Marked manuscript: Results, p. 6, line 8 to p. 13, line 21; Table 2, pp. 19-20. |
| 31 | Clarity of major themes | Four major themes are clearly identified and presented in separate Results subsections. | Marked manuscript: Results, p. 6, line 8 to p. 13, line 21; Table 2, pp. 19-20. |
| 32 | Clarity of minor themes | Nine categories and 17 subcategories, including variations in participants' experiences, are described. | Marked manuscript: Results, p. 6, line 8 to p. 13, line 21; Table 2, pp. 19-20. |

**COREQ reference**

Tong A, Sainsbury P, Craig J. Consolidated criteria for reporting qualitative research (COREQ): a 32-item checklist for interviews and focus groups. International Journal for Quality in Health Care. 2007;19(6):349-357. https://doi.org/10.1093/intqhc/mzm042
