# Supplementary material for: Climate-related psychological distress among reproductive-age women: A qualitative content analysis
Source: J Clim Chang Health. 2026 Oct 28;32:100760. doi: 10.1016/j.joclim.2026.100760 (PMC13742310; doi:10.1016/j.joclim.2026.100760)
Supplement: Supplementary file 3 [file mmc3.docx]

**SUPPLEMENTARY TABLE S1**

**Results-related supporting material**

*Additional illustrative quotations supporting the Results*

**Manuscript number:** JOCLIM-D-26-00139

**Associated article: Climate-related psychological distress among reproductive-age women: A qualitative content analysis**

**Journal:** The Journal of Climate Change and Health

The main manuscript retains one concise representative quotation for each of the 17 subcategories. Six additional quotations are presented below, ordered by theme, category, and subcategory. Participant labels are numerical study identifiers.

**Supplementary Table S1. Additional illustrative quotations supporting the Results**

| **Main theme** | **Category** | **Subcategory** | **Additional illustrative quotation** |
| --- | --- | --- | --- |
| 3.1. Living with a disrupted and increasingly insecure environment | 3.1.1. Perceived environmental disruption and insecurity | 3.1.1.1. Disrupted climatic order and intensifying heat | *“In recent years, the weather has become much hotter, and this heat has truly been unbearable for me.”* (Participant 24, 35 years) |
| 3.2. Consequences of climate change in everyday life | 3.2.1. Direct bodily and economic consequences | 3.2.1.1. Embodied consequences of heat, saline water, and polluted air | *“Polluted air and suspended particles have caused respiratory problems and allergies for us, our children, and even for the next generation. Living in this kind of climate will be more difficult for them.”* (Participant 9, 45 years) |
|  |  | 3.2.1.2. Household costs and livelihood pressure | *“Heat and reduced rainfall have made the water in Qom more saline, and this has caused skin problems and additional costs for us.”* (Participant 1, 24 years) |
| **3.3. Climate-related psychological distress** | **3.3.1. Emotional manifestations of psychological distress** | **3.3.1.2. Heat-related mood changes and reduced social tolerance** | *“Excessive heat and high temperatures make me irritable, restless, and low-spirited.” (Participant 17, 39 years)* |
|  | **3.3.2. Cognitive manifestations of psychological distress** | **3.3.2.1. Reduced precipitation as a warning of future hardship** | *“Because it has not snowed in Qom this year, I have been constantly worried that, in the summer, as in previous years, we may face water and electricity rationing and may not be able to turn on the cooler. I am very worried about how I will endure the heat.” (Participant 12, 23 years)* |
|  | **3.3.3. Moral manifestations of psychological distress** | **3.3.3.1. Moral self-monitoring during household water use** | **“Whenever I use water, I keep asking myself: Am I using too much water? Is this amount wasted? Even for simple tasks, such as washing something or performing ablution, I am constantly worried that my use of water may contribute to greater water scarcity.” (Participant 18, 45 years)** |

**Quotation notes.** Quotation wording reproduces the English translation used in the original submitted manuscript. Participant identifiers are reported in the same format as in the main text.
